# A Rare Case of Cluster Headache Occurring Exclusively During Sleep Without Autonomic Symptoms and Agitation: A Case Report and Literature Review

**DOI:** 10.7759/cureus.39021

**Published:** 2023-05-15

**Authors:** Yasutaka Sadamoto

**Affiliations:** 1 Headache Center (Neurosurgery), Takanoko Hospital, Matsuyama, JPN

**Keywords:** subcutaneous injection, sumatriptan, hypothalamus, sleep headache, agitaion, autonomic symptom, primary headache disorder, cluster headache

## Abstract

As a primary headache disorder, cluster headache (CH) is a severe unilateral headache that recurs at certain times of the year, such as during season changes. It is characterized by autonomic symptoms, such as ipsilateral lacrimal and nasal discharge, as well as an inability to stay still during headache attacks. We report a rare case of CH in a 67-year-old male who presented with a severe right-sided headache lasting 30 minutes to one hour and occurring only during sleep. The headache resolved within five minutes after the subcutaneous injection of sumatriptan and was not accompanied by any autonomic symptoms or clear agitation.

## Introduction

Repeated, brief occurrences of intense, unilateral headaches with accompanying autonomic symptoms and excitability are hallmarks of a primary headache disease known as cluster headache (CH), which probably results from an abnormality in the hypothalamus with subsequent trigeminovascular activation [1]. Subcutaneous sumatriptan injection is a special treatment option [2]. These headaches are rarely observed without autonomic symptoms [3-5]. In this report, we present a case of CH with repeated headache attacks of the circadian rhythm with an onset during sleep only and without autonomic symptoms or clear agitation. Because circadian rhythm and agitation indicate hypothalamic involvement [1], we considered this case to have limited association with the hypothalamus. We hope that this study will help to elucidate the hypothalamic connection in CH.

## Case presentation

The patient was a 67-year-old Japanese man with no recorded specific medical or family history. He reported usually going to bed at approximately 11:00 p.m. and sleeping consistently for approximately seven hours. Since the age of 55 years, he had experienced a headache attack once every two years in the early spring for approximately one month; the headache occurred only at approximately 2:00 a.m., and never during the daytime. In the past, he had visited another hospital for this headache, but no abnormality had been found on head MRI or blood tests; he had been prescribed nonsteroidal anti-inflammatory drugs (NSAIDs) such as loxoprofen, but those had proven to be ineffective.

In early March 2023, without any particular trigger, he awoke at approximately 2:00 a.m., with a severe headache centered around the right periorbital area. On a numerical analog scale of 1-10, the pain was extremely severe, reaching an intensity of 7-8. There were no accompanying symptoms such as nausea or dizziness, and the patient was unaware of any autonomic symptoms, such as tear flow, nasal obstruction, or nasal discharge on the side where the headache was felt. Later, when the patient's wife checked his face during a headache attack, she did not observe any autonomic symptoms. During the headache attacks, he did not want to lie down; therefore, he got off the bed and tried to read sitting in a chair, hoping that the headache would subside. He could not concentrate on reading because of the headache, and he did not walk around or maintain a sitting position. He experienced a strong headache but did not feel agitated. The headache lasted 30 minutes to one hour and recurred every night, and hence he visited our department on the fourth day after the onset.

On examination, no obvious neurological abnormalities were observed, and a plain MRI did not show any unusual results. Blood tests showed no inflammatory reaction, and his liver and kidney function parameters and blood glucose levels were within normal ranges. We initially considered hypnic headache (HH) and suggested drinking coffee before sleep and having coffee and indomethacin during headaches. After both of them were found ineffective, we considered migraine and atypical CH and instructed the patient to take verapamil 240 mg/day and sumatriptan nasal spray when he experienced a headache. Sumatriptan nasal spray was effective within 15 minutes, and sumatriptan subcutaneous injection was prescribed next, which turned out to be effective within five minutes, and the patient was continued on verapamil, and sumatriptan injection was used for his headaches. The frequency of headache attacks, which had been recurring every night earlier, reduced to about three times a week by the end of March and they stopped appearing altogether by April. Therefore, we reduced the dose of verapamil to 120 mg/day, but the headaches did not recur, and the patient has been off verapamil since mid-April.

## Discussion

**Figure 1:**
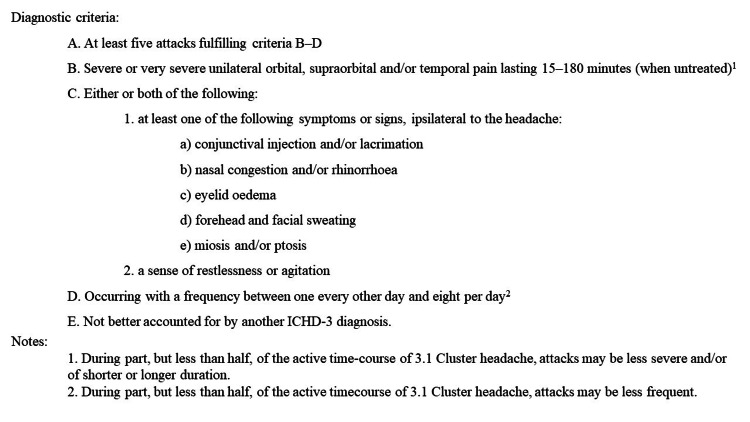
Diagnostic criteria for cluster headache Adapted with permission from the International Classification of Headache Disorders, Third Edition [8]

CH is a primary headache disorder affecting up to 0.1-2.4% of the population [6,7]. According to the International Headache Society (IHS) criteria, laid out in the International Classification Headache Disorder, Third Edition (ICHD-3) [8], CH is more common in males and is characterized by untreated severe unilateral orbital, supraorbital, or temporal headaches lasting 15-80 minutes with at least one autonomic symptom ipsilateral to the pain or agitation or both [8]. The diagnostic criteria for CH are shown in Figure 1.

Cases of CH without autonomic symptoms are rare [3-5]. The case reports of CH patients without autonomic symptoms in the literature are summarized in Table. 1. Unlike the present case, all of the previously reported CH cases without autonomic symptoms showed agitation during headache attacks [3-5].

**Table 1:** Studies on cluster headaches without autonomic symptoms published to date

Author and year	Percentage and number of without local autonomic symptoms
Ekbom, 1990 [3]	3.1% (5/163)
Nappi et al., 1992 [4]	2.8% (7/251)
Vigl et al., 2001 [5]	One (case report)

This patient had a severe unilateral headache with a circadian rhythm during sleep, which resolved without treatment within an hour. It responded rapidly to a subcutaneous injection of sumatriptan and was most likely a CH. However, it was without any autonomic symptoms or apparent agitation. Recurrent unilateral headache during sleep in major primary headache disorders includes migraine, trigeminal autonomic cephalalgia (TACs) (including CH), and HH [8]. In the context of these diseases, the present case was particularly difficult to differentiate from migraine and HH.

The differences between the present case and migraine were found in terms of the response time to sumatriptan subcutaneous injection, duration of headache, and concomitant symptoms of headache. Sumatriptan is a potent and selective agonist 5-hydroxytryptamine (5HT) 1B/D receptor, which inhibits the presynaptic release of calcitonin gene-related peptide (CGRP) following activation of the trigeminovascular system [6]. The subcutaneous injection of sumatriptan is considered a specific treatment for CH [2]. It has been demonstrated that CGRP increases blood concentration during migraine headache attacks [9], and the same increase has been observed in patients with CH [10]. The involvement of CGRP has been suggested in CH and migraine [11], and anti-CGRP monoclonal antibodies have been used clinically [12]. In clinical trials, the subcutaneous injection of sumatriptan was shown to relieve headaches within one to two hours in patients with migraine and within 15 minutes in patients with CH [13], with more immediate relief observed in patients with CH [14]. Functional impairment improves within 0.5-5 minutes after subcutaneous administration in patients with CH [15]. In preclinical studies, sumatriptan was observed to accumulate in the brain regions relevant to the migraine and CH pathogeneses, such as the hypothalamus and brainstem, as soon as one to five minutes after the injection [14]. To our knowledge, there are no diseases, such as CH, for which the subcutaneous injection of sumatriptan is effective for a very short period of time. This patient had severe headache attacks lasting 30 minutes to one hour, whereas migraine patients had headache attacks lasting about 4-72 hours [16], and he lacked the accompanying symptoms, such as nausea and light sensitivity, which are often accompanied by migraine [16].

Excluding CH, triptans are usually ineffective in TACs [17]. Major TACs excluding CH have the following characteristics and differ from the present case [17]: paroxysmal hemicrania, which is a severe unilateral headache with autonomic symptoms lasting 2-30 minutes that occurs several times a day, for which indomethacin is effective [17]; short-lasting unilateral neuralgiform headache attacks characterized by unilateral attacks of moderate-to-severe headache lasting from a few seconds to several minutes, accompanied by marked ipsilateral tearing and redness of the eye [17]; hemicrania continua, a persistent and strictly unilateral headache with ipsilateral autonomic symptoms that may be accompanied by excitement. It persisted for more than three months, and indomethacin was effective [17].

HH is rare, ranging from 0.08 to 0.22% of cases [7,18]. It is associated with the hypothalamus, similar to migraine and CH, and most headache attacks occur between 2:00 a.m. and 4:00 a.m., with one or two attacks occurring per night. More than one-third of the patients experience severe headaches, and one-third report having unilateral headache attacks [19]. Restlessness has not been observed in patients with HH [19], which, in many respects, is consistent with the present case. The most common acute and prophylactic medication is caffeine, and indomethacin is effective in treating HH [20]. For acute treatment, triptans are usually not effective for HH treatment [20]. The present case differs from HH in that indomethacin and caffeine were ineffective, whereas triptans were effective. There is only one case report of an HH responding to triptan, and it involved a 71-year-old woman with HH whose headache disappeared within 30 minutes of taking triptan (rizatriptan) after caffeine and indomethacin were found to be ineffective [15]. CH is more prevalent than HH, and the IHS diagnostic criteria for HH state that it occurs >10 days/month for >3 months [8]. The patient responded well to triptans, generally considered ineffective in HH, and had no headache attacks for over a month.

Although we were not able to confirm a favorable response to hyperbaric oxygen administration, which is another clinical feature of CH, we considered this patient to have probable CH for the abovementioned reasons. The pathophysiology of CH remains unclear. Currently, the involvement of three main factors is suggested: (1) headache involving the trigeminal pathways; (2) autonomic symptoms involving parasympathetic activation, and (3) circadian rhythms and agitation involving the hypothalamus [6,21]. The activation of the trigeminocervical complex in patients with CH has not been detected using functional imaging [22], and further studies are required to clarify the role of the trigeminovascular system in the pathophysiology of CH. Cranial autonomic symptoms arise from reflexive activation of the trigeminal-autonomic reflex pathway through parasympathetic outflow from the superior salivatory nucleus and cranial facial nerve through the sphenopalatine ganglion, resulting in vasodilatation and parasympathetic activation [10]. The presence of CH without autonomic symptoms has been previously reported [3-5], suggesting that parasympathetic activation is a consequence, rather than a cause, of trigeminal activation [23]. The maintenance of circadian processes is significantly aided by the hypothalamic suprachiasmatic nucleus [23]. It receives sensory information from the retina, enabling the biological clock to be synchronized with the light-dark cycle [23]. Orexins (hypocretins), which are synthesized in the hypothalamus, have drawn increasing attention, owing to their association with the clinical characteristics of CH, including agitation and restlessness [23]. Orexinergic neurons receive input not only from the suprachiasmatic nucleus for circadian regulation but also from areas mediating stress, emotions, and autonomic responses [23]. Studies involving positron emission tomography [24] and functional MRI [25] have also suggested the role of the hypothalamus in the pathogenesis of CH. The source of CH is thought to be the central nervous system [6,21,23]. However, there have been case reports describing characteristics of CH with autonomic symptoms that were not accompanied by excitement, suggesting that some cases of CH may not be associated with the hypothalamus [26].

## Conclusions

We reported a case of a patient who experienced recurrent headache attacks with a circadian rhythm, without autonomic symptoms or clear agitation. We believe that it was unlikely that the orexin nervous system was involved in this case. Since the hypothalamus is significantly related to the pathogenesis of CH, migraine, and HH, detailed studies of atypical CH, such as the present report, will contribute to elucidating the pathogenesis of primary headaches related to the hypothalamus.
